# Community perspectives on COVID-19 outbreak and public health: Inuit positive protective pathways and lessons for Indigenous public health theory

**DOI:** 10.17269/s41997-024-00863-z

**Published:** 2024-04-23

**Authors:** Gwen K. Healey Akearok, Zoha Rana

**Affiliations:** Qaujigiartiit Health Research Centre, Iqaluit, Nunavut Canada

**Keywords:** Inuit, Community-based participatory research, Inuit research, Public health, COVID-19, Nunavut, Arctic, Community health, Inuit Qaujimajatuqangit, Inuit, recherche participative basée sur la communauté, recherche inuite, santé publique, COVID-19, Nunavut, Arctique, santé communautaire, Inuit Qaujimajatuqangit

## Abstract

**Objectives:**

Indigenous public health theory and the voices of Canadian Indigenous communities remain under-represented in the literature despite the Canadian Truth and Reconciliation Calls to Action, and the perspectives of Inuit are further under-represented in this literature. The goal of this paper is to explore the perspectives of *Iqalungmiut* (people of Iqaluit), frontline staff, and decision-makers on the management of the COVID-19 outbreak in Iqaluit in April to June 2021 and to identify lessons learned and contributions to public health policy and practice specific to Inuit populations in Canada.

**Methods:**

This study used the *Piliriqatigiinniq* Community Health Research Model which was developed by Nunavummiut to guide community-based health and well-being research. Interviews were conducted with 44 individuals: 22 community members and shelter users; 17 frontline workers; and 5 decision-makers representing municipal and territorial government. Participants were asked about their experiences during the outbreak, sources of information, and strengths and challenges during outbreak management.

**Results:**

Challenges included overcrowding, physical disconnection from family members, and mental health and trauma. Community-identified strengths included strong interagency cooperation, food hamper and COVID-19 care kit deliveries, and travel restrictions. Several Inuit positive health-protective pathways were identified including *Ilaginniq*; *Silativut*; *Inuuqatigiittiarniq*; *Piliriqatigiinniq*; *Ikajurniq*; and *Pijitsirniq*.

**Conclusion:**

Outbreaks of infectious illness are not new to Nunavut communities and Inuit protective pathways have and continue to be critical avenues to adapt to and mitigate such challenges. This exploratory study provides clear direction for Inuit public health policy and practice in Canada, while contributing to the body of literature on Indigenous public health theory.

## Résumé

**Objectifs:**

La théorie de la santé publique autochtone et les voix des communautés autochtones canadiennes sont encore sous-représentées dans la littérature scientifique malgré les Appels à l’action de la Commission de vérité et réconciliation du Canada; les points de vue des Inuits sont encore moins représentés dans cette littérature. Notre article vise à explorer les points de vue des *Iqalungmiut* (les habitantes et les habitants d’Iqaluit), des personnels de première ligne et des décideurs et décideuses au sujet de la gestion de l’éclosion de COVID-19 à Iqaluit entre avril et juin 2021, ainsi qu’à recenser les leçons apprises et les contributions aux politiques et aux pratiques de santé publique propres aux populations inuites du Canada.

**Méthode:**

L’étude a fait appel au modèle de recherche en santé communautaire *Piliriqatigiinniq*, élaboré par des Nunavummiut pour orienter la recherche sur la santé et le bien-être des communautés. Des entretiens ont été menés auprès de 44 personnes : 22 résidentes et résidents et utilisateurs et utilisatrices de refuges; 17 intervenantes et intervenants de première ligne; et 5 décideurs et décideuses représentant l’administration municipale et le gouvernement territorial. Ces personnes ont été interrogées au sujet de leurs expériences pendant l’éclosion, de leurs sources d’informations et de leurs forces et leurs limites durant la gestion de l’éclosion.

**Résultats:**

Les difficultés rencontrées ont été l’entassement, la séparation physique des autres membres de la famille, les problèmes de santé mentale et les traumatismes. Les forces citées par la communauté ont été la solidité de la coopération interservices, les livraisons de paniers alimentaires et de trousses de soins de la COVID-19, et les restrictions des déplacements. Plusieurs voies inuites de protection de la santé ont été recensées : *Ilaginniq*; *Silativut*; *Inuuqatigiittiarniq*; *Piliriqatigiinniq*; *Ikajurniq* et *Pijitsirniq*.

**Conclusion:**

Les éclosions de maladies infectieuses n’ont rien de nouveau pour les communautés du Nunavut, et les voies protectrices inuites continuent d’être des moyens essentiels pour ces communautés d’aplanir les difficultés de la sorte et de s’y adapter. Notre étude préliminaire fournit une orientation claire pour les politiques et les pratiques de santé publique inuites au Canada tout en contribuant au corpus de recherche sur la théorie de la santé publique autochtone.

## Introduction

Indigenous public health models are frameworks and approaches that prioritize the health and well-being of Indigenous communities. These models are rooted in Indigenous knowledge, culture, and traditions, and they recognize the unique needs and strengths of Indigenous populations (Churchill et al., 2020; Lavoie & Dwyer, 2016; Lys, 2018; Pollock et al., 2018). Indigenous public health models also prioritize community engagement and participation. The Truth and Reconciliation Calls to Action call upon Canadian communities and institutions to ensure greater integration of Indigenous worldviews and perspectives into health systems and teaching materials (specifically actions 18, 19, 21, 22, 23) (Truth and Reconciliation Commission of Canada (TRCCan), 2015). The significance of public health theory lies in its ability to challenge dominant Western-centric perspectives and centre Indigenous voices, experiences, and ways of knowing. It calls for Indigenous communities to regain agency and control over health and well-being, ensuring that interventions and approaches are relevant, effective, and sustainable.

Indigenous public health models vary across different Indigenous cultures and regions, reflecting the diversity and uniqueness of Indigenous communities worldwide. The COVID-19 pandemic provided an opportunity to explore such public health in action, document experiences, and inform and broaden Indigenous public health theories and practice. Within this limited body of literature, northern and Inuit perspectives and voices are under-represented (Inuit Tapiriit Kanatami (ITK), 2014; Tagalik, 2018). The goal of this paper is to articulate the experience of one Inuit community in Nunavut, Canada, during a COVID-19 outbreak, and the Inuit caregiving pathways that were protective of public health. This research is a needed contribution to Indigenous public health theory, as well as the decolonization of public health systems and the integration of Indigenous knowledge systems and practices into policy, research, and practice.

## Context

Iqaluit is the capital city of Nunavut, the northernmost capital city in Canada. Iqaluit remained free of COVID-19 a year into the global pandemic. This was credited to several public health measures, including an immediate ban on unnecessary travel (by air or sea) and mandatory 14-day isolation protocols for necessary travel into the territory which were instituted in March 2020. The remoteness of Nunavut’s communities provided both advantages and disadvantages for the management of COVID-19. For example, remoteness of Nunavut’s 25 communities and the lack of inter-community road infrastructure provided opportunities to limit spread by limiting inter-community air travel. Disadvantages of Nunavut’s remote geography included an already limited healthcare system infrastructure and low staffing rates, as well as additional complex logistics for the disbursement of personal protective equipment and vaccines. The factors involved in COVID-19 decision-making for the territory of Nunavut at the time also included the following (Government of Nunavut, 2021): status of transmission of COVID in territory; state of testing capacity; health system capacity; transmission and case levels in gateway cities (Ottawa, Montreal, Winnipeg, Edmonton, Yellowknife); current health/medical evidence; assessment of risks and vulnerabilities; and epidemiological and public health evidence. Co-ordinated COVID-19 vaccination programs throughout Nunavut began in January 2021, prioritizing Elders and vulnerable populations (Government of Nunavut, 2021; National Advisory Committee on Immunization (NACI), 2021).

Outbreaks of infectious respiratory illness were not a new phenomenon in Nunavut communities. Influenza outbreaks in the living memory of Elders (Government of Nunavut, 2016; Sandiford Grygier, 1994; Tester & Kulchyski, 1994) and the historical and ongoing impacts of tuberculosis outbreaks in Nunavut provided a foundation from which to build a public health response (Kilabuk et al., 2019; Orr, 2013; Pease et al., 2019).

In April 2021, an outbreak was declared in Iqaluit. The number of cases grew rapidly, and by the end of May, more than 200 *Iqalungmiut*^1^ had contracted COVID-19. This was a significant number for a population of approximately 7400 individuals (Statistics Canada, 2021a). The purpose of this case study was to explore a diversity of perspectives on the outbreak response in Iqaluit for the purposes of informing future public health actions.

### Location and population demographics

Nunavut is a Canadian territory, formed in 1999 as a result of Canada’s Nunavut Act (Government of Canada, 1993). The population of Nunavut in the 2021 Canadian Census was 36,858 (Statistics Canada, 2021b) and the population of Iqaluit was recorded as 7429 in the 2016 Canadian Census (Statistics Canada, 2021a), of whom 50% self-reported as Inuit. The mean age of *Iqalungmiut* in 2016 was 32 years of age and 48% of the population were under the age of 30 (Statistics Canada, 2021a).

### Implementation of public health measures for outbreak management

The announcement of the COVID-19 outbreak on April 15, 2021, led to a lockdown in Iqaluit, with the closure of schools, childcare facilities, and most businesses. Over the course of the outbreak, the Canadian government also sent extra resources to the territory to help with the response, including healthcare workers, vaccines, and additional funding (Pemik, 2020; Ritchot, 2022).

**Fig. 1:**
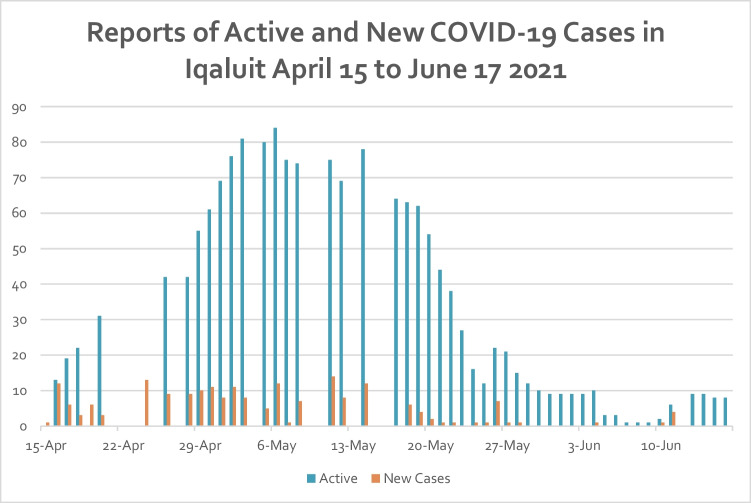
Reports of active and new COVID-19 cases in Iqaluit, Nunavut, April to June 2021

Restrictions eased slightly by June 3, and further by June 11 (see Fig. 1 for case reports). A travel exemption for fully vaccinated travelers began June 14, where fully vaccinated travelers could apply for an exemption from mandatory 14-day isolation in a designated gateway city.

## Methods

This study was initiated at the request of community members and community-serving organizations. The study was conducted by community researchers. The specific objectives of this study included (1) to explore the perspectives of *Iqalungmiut* (community members), frontline staff, and decision-makers on the management and reaction to the outbreak in Iqaluit in April to June 2021, and (2) to identify Inuit positive protective pathways to inform public health theory, policy, and practice in relation to Truth and Reconciliation Calls to Action (TRCCan, 2015).

### Study framework

This study used the *Piliriqatigiinniq* Community Health Research Model (Healey & Tagak, 2014), which was developed by Nunavummiut to guide community-based health and well-being research. The model prioritizes 5 Inuit concepts for research excellence: *Inuuqatigiitiarniq* (respect for all/shared humanity), *Pittiarniq* (to be good or kind), *Iqqaumaqatigiinniq* (to think deeply until realization), *Unikkaaqatigiinniq* (the power, meaning, and role of story), *Piliriqatigiinniq* (to work for the common good). These values provide the foundation for the research study: the study addresses key community research questions; the narratives and voices of Nunavummiut are essential to the story; the analyses focused on understanding experiences and identifying solution-seeking pathways; the study was implemented with compassion and kindness at the heart of the work.

### Data collection and analyses

Interview requests were made via email to frontline workers, decision-makers, and those working in Iqaluit’s shelter and food centre system to purposefully sample individuals who could speak to the impact of the outbreak on the community’s most vulnerable. This was a key area of importance identified by community members. Interviews with clients of the shelter system and food centre followed a snowball sampling strategy. Interviews were conducted in a comfortable setting chosen by the participant, recorded with permission, and transcribed verbatim. Interviews were conducted between October and December 2021. All questions were asked in English, and participants primarily responded in English. In the cases where they responded in Inuktitut, the authors provided the translation and verified the translation with a third party. A rigorous, respectful, and mindful process was followed for the data analysis, which included the comparison of findings to the known literature on the topic; reflexivity and bracketing of researcher perspectives before and during the study; an iterative data collection and analysis process; discussion of findings with the local Nunavut-based advisors when and where appropriate; and honouring the stories, shared by parents, by keeping their words intact as often as possible in the presentation of results without breaching confidentiality.

Interviews were conducted with 44 individuals: 22 community members and shelter users (*n* = 22 self-identified Inuit); 17 frontline and/or essential workers (*n* = 5 self-identified Inuit, *n* = 3 long-term community members of 10+ years); and 5 decision-makers representing municipal and territorial government (0 self-identified Inuit, 2 long-term community members of 10+ years, all permanent (not locum) employees). Participants were asked about their experiences during the outbreak, their sources of information, and strengths and challenges experienced during the lockdowns. An analytical approach building on the concept of *Iqqaumaqatigiiniq* (“all knowing coming into one”), which is similar to “immersion and crystallization” (Borkan, 1999), was used to identify story elements, groupings, or themes in the data. The stories shared by participants are honoured, keeping their words intact as often as possible in the presentation of results.

## Findings

Findings are focused on the following themes: initial reaction of fear and anxiety to COVID-19 outbreak; sources of information throughout the COVID-19 outbreak; strengths and challenges experienced during the outbreak; and Inuit positive protective pathways.

### Fear and anxiety

Initial reactions among community members and service providers included reports of increased levels of fear and anxiety.

*“It’s something you’ve never heard of – something you’ve never even thought about, you know? I never thought I’d go through a pandemic. I didn’t even know what a pandemic was until COVID-19 hit…. People thought it was the end of the world…”* – Community member 0025

In the context of the outbreak and infectious illness histories of Nunavut communities, expressions of fear and anxiety were not unexpected. Community members expressed the following:

- Fear of getting infected: Feeling anxious about catching the virus and becoming seriously ill, and not having access to adequate or life-saving healthcare.
- Fear of spreading the virus: Participants worried about spreading the virus to their loved ones or other community members, particularly Elders, even if they did not have any symptoms.
- Fear of death: The pandemic has led to a significant number of deaths worldwide, and participants expressed fear and anxiety about their own mortality.
- Fear of uncertainty: The constantly evolving nature of the pandemic led to feelings of uncertainty, which caused anxiety and stress.
- Fear of social isolation: Social distancing measures and lockdowns led to feelings of isolation and loneliness for some.
- Fear of child apprehension: Participants, particularly single parents and shelter users, feared that their children would be apprehended and put in the care of social services if they contracted COVID-19 and were not able to care for them.
- Fear of food insecurity: Many participants shared concerns about how they were going to provide enough food for their families and extended families and worried about having enough to eat.

Overall, fear and anxiety during the COVID-19 pandemic were normal responses, globally, to an unprecedented event, which ultimately catalyzed community action, leadership, and responses to the pandemic through formal and informal channels.

*“What are we going to do about it?” …That was the first thought and then obviously there were quite a few people that were like, “We were expecting this.” We couldn’t hide from it forever. It was bound to come here. And that’s just what I had to remind myself - it is what it is. We have to continuously be safe, protect the little ones, shut down if you have to…because we are such a vulnerable city and that’s what worries me…* – Frontline service provider 0057

### Sources of information about COVID-19 and the Iqaluit outbreak response

The Government of Nunavut provided daily briefings from the Legislative Assembly of Nunavut, which were led either by the Premier or a Minister in the Government and the Chief Public Health Officer or a designate. These briefings were broadcast over CBC Nunavut radio and social media channels, on the cable channel for the legislature, and through Government of Nunavut social media channels. These briefings provided the primary sources of information for study participants and were very highly regarded as a reliable and helpful source of information about cases, restrictions, variations on protocols, and recommendations for Nunavummiut. Examples of correct or helpful information that participants found on social media included instructions for making masks and wearing them properly, updated daily case counts for different communities and territory-wide, recommendations for social distancing, and ways to ask for and receive help during the lockdown periods.

Other sources of information identified by participants included family and friends, and CBC North Radio and Television. Shelter users identified nurses conducting contact tracing in the community as an important source of information about the outbreak, as they were not able to access the other methods of communication.

### Community-identified challenges during the outbreak

*“We stopped going out ski-doing or ice fishing with people. We eat with our hands, so it was weird… And so I kind of had a change like every part of me… I was like what do I do, what do I do? And I started freezing. I’m not sure how this works now? Do I panic? Do I not panic? Is it okay? And I was like oh I can actually be with my family. I just really tightened my circle and went ‘okay, this is enough’. And then - my husband and the kids I remind them all the time - it’s about us… COVID-19 did bring out issues in my heart about our Elders passing away. And Elders around the world.”* – Community member 0023

Participants in this study shared the challenges they both experienced and observed during the outbreak, as well as their own strategies for mitigating the challenges. Participants highlighted challenges that were a result of the physical disconnection from family members; living in crowded homes; the partying crowd in Iqaluit; and ties to mental health and trauma which are elaborated upon below.

#### No hugging, no gathering, no social support

Participants referred to the lack of hugging with such frequency that it arose as a theme in the data. The elimination of in-person social connectivity was difficult, especially because Inuit society is based on kinships (Arnakak, 2000).

*“no hugging is hard and being distant. That that part is hard…I saw my father at church yesterday and not being able to hug him, that was hard. Well, I haven’t gone to church for over a year. I finally go … and not being able to hug him. The last time I seen him was over a year. And when I got home, I got so emotional. And I didn’t know where it was coming from.”* – Shelter client 0022

#### Overcrowding

*“Housing, housing [is] a challenge. There’s a lot of over-crowding…housing has always been an issue in Iqaluit and Nunavut. There are places that are full. I came across a family – one of my cousin’s friends, there’s like 10 to 15 people in the same household. Yeah, lots of kids.”* – Community member 0029

Participants highlighted the ways in which overcrowding amplified the impacts of the COVID-19 outbreak in the community. The lack of adequate space to distance, the lack of places to sleep, the lack of quiet spaces for students to study, and the lack of personal space as key issues for themselves and other community members.

*“We have people who, you know, who might have 8 to 10 to 12 people living in a two bedroom apartment. So, housing is the biggest issue here … So, if you had a family from Nunavut, it has like a husband and wife or partner, and they’ve got five kids and they have their own home, [they are fine]. But when you’ve got people all packed in together … you don’t know. And it’s the same thing for TB. Again, even that wouldn’t be as bad as it is if everyone could have their own home here… that is such a big thing that to me. It will make a difference for any next disease or any pandemic or anything else.”* – Community member 0028

Participants in this study also highlighted the challenges of large families in small living quarters and the impact of isolating with an individual who was violent or unsafe in that context. Participants noted solutions such as providing alternate accommodations for isolating individuals, and more investment in single family homes.

*“I think the biggest lesson, well for me, is a stay at home order only really works for people in positions of a certain amount of privilege. For a lot of people, a stay at home order is quite awful, isolating in a home that may not be safe or just does not have space for the people in there. It’s so limiting to some people’s, economy and opportunities to have to just be at home… We have to, I think, any future public health measures that require some form of isolation, I think you need to consider some form of alternates like reasonable accommodations.”* – Frontline service provider 0034

#### Parties, addictions, mental health, and trauma

A very rare and uncommon response from study participants involved a discussion about “party” and drinking activities in Iqaluit; however, this perspective is important for public health and thus was included as a finding.

*“No, [COVID-19 didn’t] stop me from seeing people…I don’t think – honestly, I didn’t really follow the restrictions all that well. Because I’d be out there partying with a whole bunch of people and then I’d go out … Normal for me.”* – Shelter client 0021

The few participants who openly disclosed that they would consume alcohol and cannabis and continue to attend parties, socialize, and gamble, rather than follow COVID-19 restrictions were users of the shelter system, had multiple stressors in their lives, and had no permanent, safe residence.

A number of participants talked about their mental health and the mental health of those around them. Mental health was a unifying theme across community members, frontline providers, and decision-makers—both their own mental health and the mental health of the community. Community members discussed how the pandemic caused trauma to resurface and highlighted how they moved through their own coping processes to mitigate the impact of the resurfaced trauma in their lives.

*“Because I know some things that have brought back people’s past trauma of somebody outside coming in to get us. And it was an invisible thing, like my family felt that as well …intergenerational trauma. And I think the whole world deals with that on the whole. Traumas get resurfaced… You go back to you know introspection and working on issues of our own and moving forward from there… I’ve also started taking off and going on the land by myself. Which I was not doing before. And then just sitting - and even if I’m sitting in the car and say oh wow the world is so beautiful. I say that I love myself, I love the world, and everyone and then I just – I almost fell apart … and then put it back together in a different way. And it was better.”* – Shelter client 0023

### Community-identified strengths during the outbreak

#### Interagency partnerships and hard-working frontline health and community-based staff

Participants largely praised the Government of Nunavut, the airlines, and the Inuit associations and community organizations that provided supports during the COVID-19 outbreak.

*“I think, how it was, in some ways, very fortunate in a lot of ways. The numbers, you didn’t see the numbers that you had seen in the south. The government seemed to be very right on top of it, regards to the [isolation] hub in Ottawa, coming in and out. People stayed put, as much as they didn’t want to stay put, they stayed put. And I think that they prevented a huge, huge loss. I do. I think the government did a great job in regards to [Iqaluit]. I think they were very fortunate in a lot of ways. Per ratio I think the numbers were far less here in Nunavut than anywhere else.”* – Frontline service provider 0037

Community members, frontline providers, and decision-makers praised the collaborative nature of the response and the interventions that were implemented, such as travel restrictions, mass COVID-19 vaccine clinics for youth, and mobile vaccination clinics to reach vulnerable neighbourhoods and individuals.

*“I think the ability to think outside the box and reach out to vulnerable populations…. there was a mobile van that drove around town, parked in more vulnerable neighbourhoods, offered vaccines, and made country food accessible for people. The testing clinic at the [church] hall, which is just a little bit more accessible for general traffic…I think there was an ability to go above and beyond and reach people. Also partnering to deliver that vaccine and mass vaccine clinics was really effective for young people and it was a strong partnership. So, I think there was just some good partnerships and thinking outside the box to really try and reach some more vulnerable populations for sure.”* – Decision-maker 0052

#### Food hampers and COVID-19 care kits

Numerous territorial, regional, and community organizations, as well as municipalities, implemented different programs to provide household support, such as food box/hamper (non-perishable items and diapers) deliveries; COVID-19 care kits (soap, cleaning supplies, masks, sanitizers, activities for children); and the delivery of country food (Inuit foods, such as seal, fish, caribou, walrus) to Elders and to isolating households. The hampers were designed to provide families with enough food to last for a period of time, typically a week or two, depending on the size of the family. Organizations typically worked with local Iqaluit businesses and suppliers to obtain the food items and received donations from individuals or groups. Food hamper deliveries were a lifeline for families who were struggling to make ends meet, especially during the Iqaluit outbreak when many people experienced reduced income. They were most helpful for families who were unable to leave their homes due to quarantine or isolation requirements. Formal responses, through governments or organizations, and informal community- or family-organized food sharing were identified as critical supports in the community. Interestingly, food hamper interventions were also very successful in the Northwest Territories and Yukon.

#### Travel ban (“keep the virus out”)

A travel ban, also known as travel restrictions, was a measure put in place by the Government of Nunavut to limit or prevent people from traveling to the territory during the COVID-19 pandemic. This restriction was designed to help control the spread of the virus by reducing the number of people carrying the infection from one location to another. While the Nunavut travel ban had impacts on the tourism industry and the ability of people to travel for work or to see family and friends, it was still widely supported by Iqalungmiut.

*“My biggest thing was – and I applaud the government – is they put their restrictions in with the airlines. So, [people] were not allowed to come here. For me, I couldn’t believe it so all of a sudden …[it was] a big thing in Canada …and the airlines said ‘okay, we’re shutting her down’. We’re not letting anybody get in here who’s going to harm our people. And I thought that was fantastic. And it was in like two days [after the declaration of the Public Health Emergency] and to me that made such a difference. And then they just continued. And they had to have the [isolation hubs] and you had to have a have a [travel permission] letter…you know, you couldn’t just get on the plane.”* – Frontline service provider 0028

The travel ban was an important tool in helping to control the spread of COVID-19, as it resulted in the delay of the arrival of COVID-19 into the territory to November 2020, allowing more time for preparation and resource acquisition (personal protective equipment, for example) and also for planning for upcoming vaccination programs, once the vaccine was approved (Healey Akearok 2023; Government of Nunavut, 2021).

## Inuit positive protective pathways

Through their stories and examples, participants explicitly described public health practices that were both challenging and needed, and implicitly identified a number of Inuit positive health-protective pathways that were elevated during the COVID-19 pandemic. These included the following: *Ilaginniq* Family and social networks; *Silativut* Our land/environment; *Inuuqatigiittiarniq* Compassion and respect for others; *Piliriqatigiinniq* Acting for the collective/common good; *Ikajurniq* and/or *Pijitsirniq* Helping each other and without the expectation of return.

*“There’s definitely a lot of community pulling together, either pulling together of food pantries, or fun things like radio shows and being on radio, that type of thing... I know the first thing was schools and stuff so there’s a lot of concern around schools with [food] programs no longer being accessible, and then you’ve got a lot of volunteers come together to hand out little food packages, that type of thing. Yeah in terms of the community coming together to do fun things, especially for children and vulnerable people and get them, if they need food, get them food, that type of thing.”* – Decision-maker 0055

### *Ilaginniq* Family and social networks

Inuit society is kinship-based and relationships and connections among family, extended family, adoptive family, and friend relationships are a critical positive protective pathway for health and well-being in Nunavut communities (Arnakak, 2000). Even though the lack of in-person connection was a challenge (as identified above), community members found ways to connect with and support each other through text messaging, social media, phone calls, and leaving messages on community radio stations to be broadcast across communities. Using radio as a tool to connect across communities was an important contributor to positive mental health and maintaining relationships to family in other areas.

*“[What Iqaluit, as a community, learned from the pandemic] was to be there for each other. That’s what I really felt.”* – Shelter client 0030

### *Silativut* Our land/environment

The Inuit holistic worldview which ties health and well-being to the land and environment is well documented (Arnakak, 2000; Department of Education, 2007; Healey Akearok et al., 2018; Karetak et al., 2017; Redvers, 2016). The role of the land and access to the land in supporting mental, physical, emotional, and spiritual well-being was also highlighted in the present study. Examples included the land as a therapeutic space for coping with resurfaced or ongoing trauma; for finding space and peace to cope with anxiety and/or crowding; to maintain some physical activity and keep the body moving; for supporting relationships and connecting with others in a safe, socially distant way; and for harvesters providing needed food and resources to Elders, communities, and families facing food insecurity.

### *Inuuqatigiittiarniq* Compassion and respect for others

This value emphasizes the importance of being respectful and kind towards other people and is viewed as a key component of living in harmony with others. It emphasizes the importance of treating others with kindness and empathy, and of acting in ways that promote the well-being of the community as a whole. Participants in this study highlighted the need and desire to care for the most vulnerable during the outbreak, such as shelter users and children experiencing unsafe home situations. They also discussed the desire to prioritize Elders and praised the implementation of, for example, “Elder hours” in the stores and post office, which limited public access to such spaces to allow Elders to shop or retrieve mail without having to worry about interacting with the public.

### *Piliriqatigiinniq* Acting for the collective/common good

Inuit collaboration processes value open communication and consensus-building. Decisions were and continue to be made through a process of communication, discussion, and consultation, with all members of the community having a voice in the decision-making process. This approach emphasizes the importance of considering different perspectives and reaching a collective agreement that is acceptable to everyone. *Piliriqatigiinniq* recognizes the interdependence of individuals and the community as a whole and is seen as a way of ensuring the well-being of all. In the present study, participants highlighted the processes and pathways through which they observed collaboration, such as for the distribution of food hampers and COVID-19 care kits; support for the travel restrictions; getting vaccinated to protect others; respecting public health measures; prioritizing the needs of Elders; and showing concern for the more vulnerable members of the community, such as the homeless.

### *Ikajurniq* and/or *Pijitsirniq* Helping each other and without the expectation of return

The concept of *Pijitsirniq*, also referred to as “serving others” or “humility,” is a central value that emphasizes the importance of putting the needs of others before one’s own and acting in service to the community. It involves a deep sense of humility and a willingness to help others without seeking recognition or reward. At its core, *Pijitsirniq* is about acting with kindness, compassion, and selflessness towards others. This can take many forms, from sharing resources and helping those in need to offering support and guidance to others. In the present study, participants identified numerous practices and examples that were grounded in the concept of *pijitsirniq*, for example shoveling snow for neighbours, sharing gear and food, taking people on the land, delivering resources and gifts to homes, volunteering for different support programs, developing at-home activities for children, and harvesting and sharing catches.

*“We had two weeks to wait [in isolation] and they brought us food – we had more food than I don’t know [what to do with it]. I packed them up and then I put it at my neighbour’s door. Then I called him and said ‘We have too much food. Here you go.’”* – Community member 0023

**Fig. 2:**
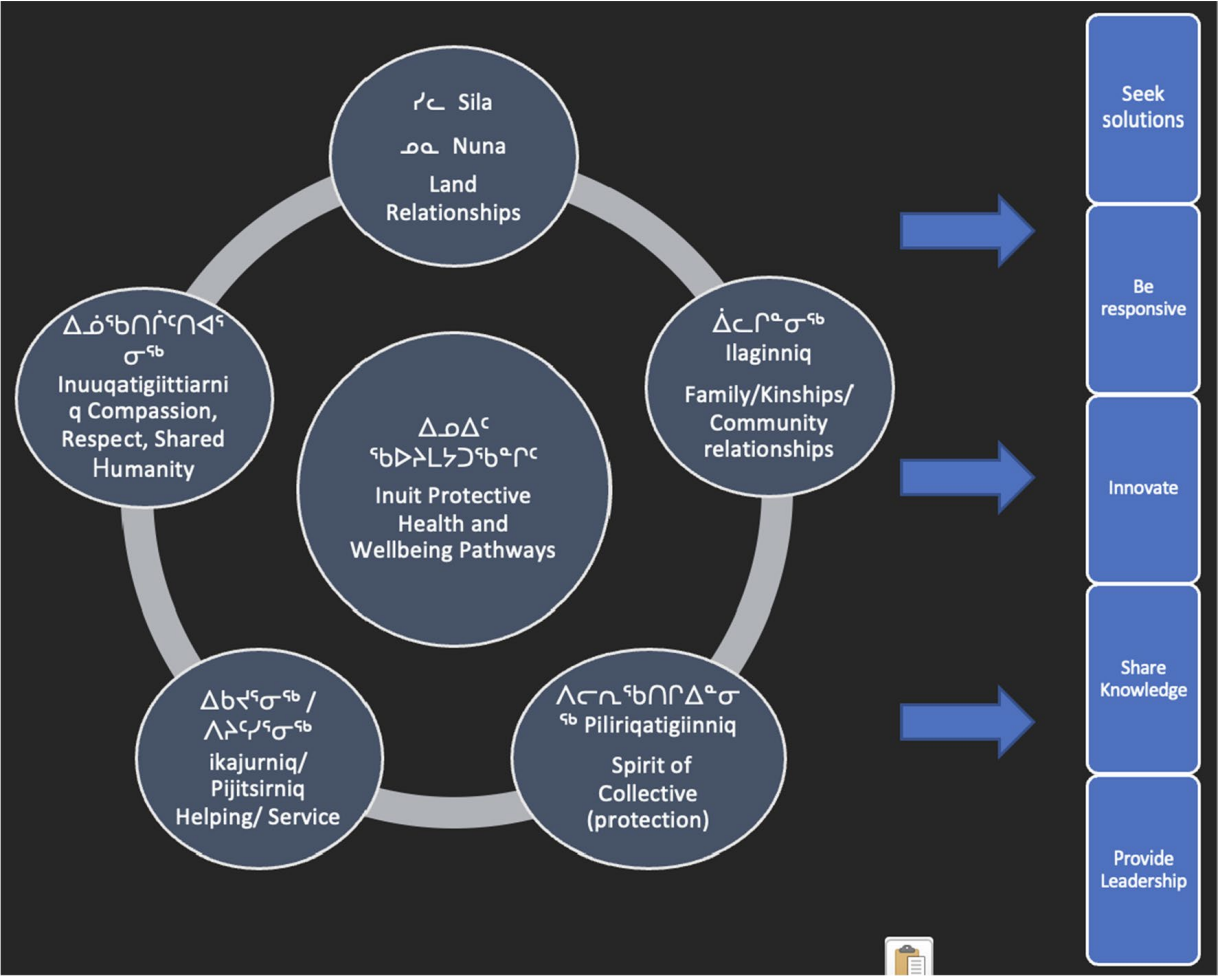
Inuit positive health-protective pathways contribute directly to formal and informal public health intervention processes

Inuit positive health-protective pathways contributed to the formal and informal implementation of COVID-19‒related interventions and adaptations at the individual, community, and regional level, by: cultivating a solution-seeking environment; emphasizing the need to be responsive to the needs of others; supporting and encouraging innovation; sharing knowledge and communicating effectively; and providing formal and informal leadership in response to a crisis (Fig. 2).

## Discussion and learnings for public health

Cultural safety and humility are central principles of Indigenous public health models (National Collaborating Centre for Indigenous Health (NCCIH), 2021). They acknowledge the historical and ongoing impacts of colonization, discrimination, and systemic injustices on Indigenous health (Redvers, 2020). These models strive to create safe and respectful spaces that honour and integrate Indigenous cultures, languages, and healing practices (Healey et al., 2016; Watson et al., 2022). Decolonizing public health theory and approaches involves elevating and embracing Indigenous public health models which often incorporate healing practices and land-based approaches. They recognize the importance of connecting with the land, revitalizing traditional healing methods, and promoting cultural continuity as essential components of health promotion and disease prevention (Kovach, 2009; Watson et al., 2022).

In the present study, *Iqalungmiut* identified a number of strengths and challenges during the 2021 COVID-19 community outbreak to inform Inuit-centric public health models. Important practical learnings for public health included the following:

1. **Celebrate and build on Inuit values and practices**. Inuit values inspire leadership through a focus on community- and consensus-building. In Inuit culture, decision-making is often based on a process of dialogue and consultation, with input from all members of the community. This approach to leadership fosters a sense of shared ownership and responsibility and helps to ensure that decisions are made in the best interests of the community as a whole. Inuit values-driven actions were directly responsible for the good vaccination rates, high levels of formal and informal communication and knowledge-sharing, the prioritization of vulnerable people (Elders, homeless) during the pandemic, sharing of food and other resources, and selfless and compassionate actions of community members. These values have and continue to help Inuit communities to navigate complex challenges and find innovative solutions to problems facing public health in Nunavut.
2. **Improved and increased mental health supports**. Nunavut communities have experienced a significant amount of trauma. This trauma is linked to the history of colonization and forced assimilation that Inuit have experienced (Qikiqtani Inuit Association (QIA), 2012; Royal Commission on Aboriginal Peoples, 1996; Tester & Kulchyski, 1994), which has had long-lasting impacts including increased rates of mental health issues, substance abuse, and suicide (Mental Wellness Advisory Committee, 2002; Kirmayer et al., 2000; Marsh et al., 2015; Tester & McNicoll, 2004; Young et al., 2015). COVID-19 contributed to a sense of hopelessness and despair, which in turn exacerbated trauma and mental health issues. Participants in this study strongly recommended the need for more support for mental health, healing, and addiction recovery to effectively address a multitude of public health issues.
3. **Supports for safe isolation impacted other longstanding unresolved infectious illnesses such as tuberculosis**. The data in the present study demonstrate that the lockdowns and public health measures, such as masking, were very effective for limiting the spread of illness as evidenced by the delayed arrival of COVID-19 to the region. Such interventions have been recognized globally for their impact on other respiratory illnesses such as respiratory syncytial virus (RSV) and influenza during the same period (Britton et al., 2020; Groves et al., 2021). Early evidence suggests that during the first lockdown period in 2020, medical evacuations for infants with respiratory illness were minimal, whereas before COVID-19 public health emergency was declared, they accounted for half of all medevacs (Healey Akearok et al., 2020). Supporting families to isolate safely and with appropriate resources (such as food, sanitation supplies, masks, children’s activities, and income) was essential to ensuring adherence with the public health measures and for supporting family well-being during a challenging time. Variations on such interventions should continue to be examined for other problematic infectious illnesses in Nunavut such as tuberculosis and RSV.
4. **Addressing the underlying social determinants of health is essential**. Social determinants of Inuit health are key factors that can significantly impact an individual’s well-being as well as community health outcomes. Social determinants of health refer to the social and economic conditions in which people are born, grow, live, work, and age, and include factors such as access to education, employment, safe housing, and healthcare, as well as broader social factors such as discrimination and inequality (ITK, 2014; Kilabuk et al., 2019; Nunavut Dept. of Health and Social Services, 2005). By addressing social determinants of health, policymakers and healthcare professionals can help to reduce health disparities and promote greater health equity. Participants in this study identified a range of interventions, including improving access to healthcare and healthcare infrastructure; improving social services; addressing water infrastructure issues; adequate and safe housing to eliminate crowding; improved access to education and training; and working to address broader social and economic factors that can impact health outcomes, such as poverty, colonialism, and discrimination. Addressing Inuit social determinants of health is a critically important step to ensure greater health equity and that all Nunavummiut have the opportunity to live healthy, fulfilling lives.

## Conclusion

The COVID-19 pandemic has been a challenging and stressful time globally. Outbreaks of infectious illness are not new to Nunavut communities, and Inuit protective pathways have been and continue to be critical avenues to adapt to and mitigate such challenges. The voices of Inuit are under-represented in the public health literature and Indigenous public health theory and practice in Canada. Understanding Inuit pathways to health and well-being is essential for mobilizing Indigenous public health practices and training the next generation of public health practitioners in Canada and globally. The significance of Inuit public health knowledge and science lies in its ability to challenge dominant Western-centric perspectives and centre community voices, experiences, and ways of knowing.

This exploratory study of perspectives on the 2021 Iqaluit COVID-19 outbreak provides clear direction for public health policy and practice in Nunavut territory, and potentially other Inuit, Arctic, and/or Indigenous populations. Future research should continue to better integrate policy and program innovations that emerged from and during the COVID-19 pandemic. In Nunavut, this work should continue to be led and implemented by communities.

## Contributions to knowledge

What does this study add to existing knowledge?

- This study describes experiences of a northern Inuit community during the global pandemic and shares insight on both Inuit positive protective pathways for community well-being and traditional public health interventions. This is a rare perspective in the public health literature, which has yet to respond to the TRC Calls to Action.

What are the key implications for public health interventions, practice, or policy?

- Key voices and perspectives from Canadian Indigenous community members in the Arctic contribute to Indigenous public health theory and guide future public health decision-making.

## Researcher positioning statement

GKHA was born and raised in Iqaluit, Nunavut, and continues to live in and work in service for her home community. She is the co-founder of the Qaujigiartiit Health Research Centre in Iqaluit. ZR spent her childhood and adolescence in the NWT and Nunavut before leaving to attend school, and since returned to Iqaluit as a public health researcher at the Qaujigiartiit Health Research Centre. Both authors live and work in Iqaluit, Nunavut.

## Data Availability

Electronic copies of transcripts are available for review for data transparency purposes.

## Footnotes

1 Inuktitut language term for “people of Iqaluit”
